# Risk of All-Cause Mortality in HIV Infected Patients Is Associated with Clinical, Immunologic Predictors and the *CCR5 Δ32* Deletion

**DOI:** 10.1371/journal.pone.0022215

**Published:** 2011-07-18

**Authors:** Milosz Parczewski, Dorota Bander, Magdalena Leszczyszyn-Pynka, Anna Urbanska, Mariusz Kaczmarczyk, Andrzej Ciechanowicz, Anna Boron-Kaczmarska

**Affiliations:** 1 Department of Infectious Diseases and Hepatology, Pomeranian Medical University, Szczecin, Poland; 2 Department of Molecular Medicine, Pomeranian Medical University, Szczecin, Poland; University of Leuven, Rega Institute, Belgium

## Abstract

**Objective:**

Investigation of the interplay between the *CCR5 Δ32/wt* genotype and demographic, epidemiological, clinical and immunological factors associated with mortality in the cART era.

**Design:**

Longitudinal data from 507 HIV-infected patients following the *Δ32* allele detection were analyzed.

**Methods:**

Cumulative 15 years mortality was calculated using Kaplan-Meyer methodology. Hazard ratios were estimated using univariate Cox models. Basing on Akakie information criteria and statistical significance multivariate Cox model was constructed and effect plots presenting adjusted hazard ratio time-dependency were drawn. Analysis of the association of all-cause mortality and *CCR5 Δ32/wt* genotype prior to the antiretroviral treatment (cART) initiation (n = 507) and on the therapy (n = 422) was also performed.

**Results:**

A mortality rate of 2.66 (CI 2.57–3.19) per 100 person-years was observed. Univariate analysis factors modifying the risk of death included the *CCR5* genotype, gender, history of cART, AIDS diagnosis and also CD4 lymphocyte nadir, zenith, the latest CD4 count and stable levels >500 cells/µl. For multivariate analysis the following predictors were selected: *CCR5* genotype (HR for *wt/wt* 2.53, CI 1.16–5.53, p = 0.02), gender (HR for males 1.91, 95%CI 1.1–3.36, p = 0.023), introduction of combined antiretroviral treatment (HR 4.85, CI 3.0–7.89, if untreated or treated <1 month, p<0.0001) CD4 count of 500 cells/µl for six months or more (HR 4.16, CI 1.95–8.88 if not achieved, p = 0.028), the latest CD4 count (HR 5.44, CI 3.39–8.74 for <100 cells/µl, p<0.0001) and history of AIDS (HR 1.69, CI 1.03–2.79, p = 0.039). Among untreated individuals the *Δ32/wt* genotype was associated with notably better survival (p = 0.026), while among cART treated individuals the *Δ32* mutation did not correlate significantly with higher survival rates (p = 0.23).

**Conclusions:**

The *Δ32 CCR5* allele is associated with a reduction of the risk of all-cause mortality in HIV (+) patients alongside clinical and immunologic predictors such as AIDS, history of cART, lymphocyte CD4 cell count and gender.

## Introduction

Infection with the human immunodeficiency virus type 1 (HIV-1) requires attachment to one of the principal chemokine coreceptors, namely CCR5 and CXCR4 for effective entry into CD4+ T-cells [1]. Use of CCR5 is frequently associated with the early stages of infection while progression of human immunodeficiency virus infection to AIDS and death is related to a viral tropism switch to CXCR4 [2]. Genetic variants of the chemokine receptors and their ligands modify susceptibility to HIV infection and the course of the disease, with a 32 base pair deletion (*Δ32*) in the reading frame of the *CCR5* gene associated with reduced susceptibility to infection and delayed disease progression [3], [4]. *Δ32/Δ32* homozygotes remain resistant to infection with CCR5 tropic variants of HIV, while among heterozygous *Δ32/wild-type* (*wt*) subjects beneficial, AIDS protective effects, have been noted in the early years of infection [5], [6]. Studies of the long-term non-progression among HIV-infected patients have indicated higher frequency of this protective allele among individuals spontaneously controlling the infection [7]. This variant has also been associated with better virologic response to combined antiretroviral therapy (cART) as well as less frequent virologic failure, decreased risk of early death among perinatally infected children and protection from AIDS among antiretroviral-treated patients [8]–[11].

In the era of combined antiretroviral treatment (cART) and expected long term survival an array of interplaying factors influence mortality among people living with HIV. Lymphocyte CD4 metrics, early introduction of antiretroviral treatment and preservation of immunological function are the key contributors to the risk of mortality in this group [12], [13]. As the interaction between clinical, immunological, virologic factors and genetic variants influencing survival have not been studied so far in detail this study aimed to evaluate the impact of *CCR5 Δ32* mutation on the long-term mortality of patients living with HIV. Characteristically in Europe, as well as in Poland, most HIV cases are diagnosed late necessitating immediate initiation of combined antiretroviral treatment [14], [15]. Therefore the rationale for this analysis was that beneficial effects of the mutation are likely to be disproportionately seen in this population due to a protective effect prior to diagnosis, enhanced by a favorable effect following initiation of the antiretroviral treatment.

## Materials and Methods

### Study population

For the study longitudinal data of 507 patients followed-up from January 1996 to June 2010 at the Department of Infectious Diseases and Hepatology, Pomeranian Medical University, Szczecin, Poland and Out Patients' Clinic for Acquired Immunodeficiency, Regional Hospital, Szczecin, Poland were analyzed. The study protocol was approved by the bioethical committee of Pomeranian Medical University, Szczecin, Poland (approval number BN-001/34/04). Written informed consent was obtained from all subjects participating in the study. Time zero was defined as a date of positive screening HIV test if later confirmed by Western-blot, immunoblotting or positive serum HIV-RNA or a confirmation test itself. The following data were collected: age, gender, date of HIV diagnosis, route of transmission, hepatitis C co-infection, clinical category at diagnosis according to CDC case definition [16], date and diagnosis of AIDS events, date and reason of death, baseline HIV viral load, history of cART as well as baseline, nadir, zenith and the latest CD4 counts, duration of CD4 count >500 cells/µl. Baseline CD4 counts are defined as the first documented result after diagnosis of HIV. Data on nadir and zenith (the lowest and the highest lymphocyte CD4 count throughout the period of observation) lymphocyte CD4 counts were collected as well. The latest lymphocyte CD4 count was taken as the last recorded value prior to the end of observation or death. CDC category at diagnosis was assumed based on the review of the clinical record of the patient, in cases of late care entry with documented prior HIV test category A (asymptomatic) was assumed if no apparent immunodeficiency was reported or available from medical records. Data on chronic hepatitis B were not included into the analysis due to small number of confirmed HIV/HBV co-infection cases while the parameter of the latest HIV-RNA levels was removed as it was performed only in 60% of patients, often being collected up to one year from the date of final observation, which was related to the poor availability of the assay.

### Study endpoint

Study endpoint was defined as all-cause mortality excluding cases with documented accidental death. Determination of the reason of death was based on the following data: autopsy report (42 cases), medical record of in-hospital treatment with cause of death defined by the treating physician (32 cases), other medical report or letter (19 cases); in four cases the cause of death remained undetermined. Validation of the underlying cause of death was performed by an independent clinician not involved in the patient care with discrepancies in the data thoroughly discussed. AIDS related death was assumed if the criteria (either presumptive or definitive) outlined by the CDC case definition were met with at least one AIDS defining condition observed at the time of death. End of observation date was defined as either death date, last recorded date of visit (cases lost to follow-up) or 1^st^ of June 2010 for the patients remaining under care (termination of data collection). When it was impossible to determine the exact date of death (6 cases) the median date between the last recorded visit and information on death was assumed as the death date.

### DNA extraction and CCR5 genotyping

For genomic DNA extraction from whole blood samples QIAamp DNA Blood Mini Kit (QIAgen, Hilden, Germany) was used with re-suspended DNA stored at 4°C for further analyses. If whole blood samples were unavailable frozen serum samples were used with extraction performed using Sherlock AX kits (A&A Biotechnology, Gdynia, Poland). For the *CCR5 Δ32/wt* genotyping a previously described methodology was used [17] with reaction products electrophoresed on a 3% agarose gel (SIGMA, Saint Louis, USA) stained with DNA-star dye (Lonza Inc, Rockland, USA) and visualized under UV light.

### Statistics

Statistical analyses for nominal variables were performed with chi-square test using EPI6 Statcalc software (Department of Mathematics, University of Louisiana-Lafayette, Lafayette, LA, USA), for continuous variables, the Mann–Whitney U-test was used (Statistica software, Statsoft, Tulsa, OK, USA). Cox models for survival were used to assess the effect of analyzed parameter on the risk of death. For all analyzed parameters unadjusted (univariate) Cox models were used to calculate the hazard ratio (HR) and included *CCR5* genotype, transmission route, hepatitis C co-infection, HIV infection stage at diagnosis, history of antiretroviral treatment, baseline viral load (log HIV-RNA copies/ml), baseline CD4 count (cells/µl), nadir CD4 count (cells/µl), zenith CD4 count (cells/µl), time with CD4 count >500 cells/µl, the most recent CD4 count (cells/µl) and history of AIDS. Moreover, for every parameter Kaplan-Meyer cumulative mortality was calculated and plotted with statistical significance testing using log-rank test. To select the most informative parameters for the multivariate model Akaike information criteria (AIC) were used to assess the fit of the each model (lower AIC indicates better fit) [18]. A final multivariate model was calculated by Cox regression for the 15-year observation and effect plots presenting adjusted hazard ratio changes over time were drawn for this model. To test the proportional hazards assumption for the multivariate Cox regression model coxph function of the R package was used. P-values of <0.05 were considered significant with values in the range of 0.1–0.05 regarded as borderline statistically significant.

## Results

### Patient characteristics

In the analyzed group of 507 HIV (+) patients the *CCR5 Δ32* genotype frequency was 14.0% with *Δ32* allele frequency of 7.0%. No *Δ32/Δ32* homozygotes were observed in the group. All patients were of Caucasian ethnicity with predominance of men (n = 363, 79.5%), median baseline age of 30 years, 50.0% infected by injection drug use, median (inter quartile range - IQR) baseline CD4 count of 229 (634-518) cells/µl and median (IQR) viral load of 74000 (14222–315000) HIV-RNA copies/ml (table 1). Mean (IQR) time of observation in the group was 83 (33-129) months. AIDS was diagnosed in 193 (38.1%) cases while median (IQR) AIDS-free time was 52 (11-115) months; antiretroviral treatment was initiated in 84.5% of patients.

**Table 1:** Group characteristics by the *CCR5* genotype.

	*CCR5 Δ32/wt* genotype	*CCR5 wt/wt* genotype	total	*CCR5 Δ32/wt* genotype frequency (%)	p value
No. of patients* (%)	71 (14.0)	436 (86.0)	507 (100)	14.0	n/a
***Gender***					
female (% of total)	14 (2.76)	130 (25.64)	144 (28.4)	9.72	0.08
male (% of total)	57 (11.24)	306 (60.36)	362 (71.6)	15.7	
***Clinical category at diagnosis***					
A (% of total)	44 (8.92)	156 (31.64)	200 (40.57)	22.0	0.003
B (% of total)	14 (2.84)	164 (33.27)	178 (36.11)	7.87	
C (% of total)	13 (2.64)	102 (20.69)	115 (23.33)	11.3	
***Likely infection mode***					
MSM (% of total)	18 (3.58)	94 (18.6)	112 (22.27)	16.07	0.07
Heterosexual (% of total)	16 (3.18)	120 (23.86)	136 (27.04)	11.76	
Injection Drug Use (% of total)	36 (7.16)	218 (43.34)	254 (50.50)	14.17	
***History of cART during follow-up***					
cART >1 month (% of total)	55 (10.93)	368 (73.16)	423 (84.1)	13.0	0.1
Never on cART or <1 month (% of total)	16 (3.18)	64 (12.72)	80 (15.9)	20.0	
***History of Hepatitis C***					
HIV/HCV coinfected (% of total)	40 (8.62)	224 (48.28)	264 (56.9)	12.0	0.32
HIV monoinfection (% of total)	24 (5.17)	176 (38.93)	200 (43.1)	15.5	
***Diagnosis of AIDS during follow-up***					
AIDS diagnosed (% of total)	24 (4.73)	169 (33.33%)	193 (38.07)	12.44	0.42
AIDS-free (% of total)	47 (9.27)	267 (52.66)	314 (61.93)	14.97	
***Mortality***					
All-cause death (% of total)	7 (1.38)	86 (16.96)	93 (19.13)	7.53	0.05
Aids related death (% of total)	5 (0.98)	48 (9.46)	53 (10.45)	7.58	0.31
Non-AIDS related death (% of total)	2 (0.03)	38 (7.45)	40 (7.89)	5.0	0.07
***Immunologic and virologic characteristics***					
Median baseline lymphocyte CD4 count (IQR) (cells/µl)	280 (44–539)	209 (64–417)	229 (63.5–417.5)	n/a	0.2
Median nadir lymphocyte CD4 count (IQR) (cells/µl)	171 (41–332)	138 (47–300)	147 (46–303)	n/a	0.33
Median zenith lymphocyte CD4 count (IQR) (cells/µl)	539 (380–810)	520 (316–780)	522 (322–780)	n/a	0.42
Median baseline viral load (IQR) (copies/ml)	85200 (21600–310000)	70500 (14000–320000)	74000 (14222–315000)	n/a	0.94

n/a – non-applicable. Sample sizes vary due to data availability: *CCR5* genotype, gender, AIDS diagnosis – 507 persons, HCV co-infection status - 464 persons, transmission route and history of antiretroviral treatment – 503 persons, HIV infection stage at diagnosis – 489 persons, baseline viral load (log copies/ml) – 288 persons, baseline lymphocyte CD4 count (cells/µl) – 488 persons, nadir lymphocyte CD4 count (cells/µl) – 494 persons, zenith lymphocyte CD4 count (cells/µl) – 499 persons, time with lymphocyte CD4 count >500 cells/µl – 500 persons, latest lymphocyte CD4 count – 504 persons.

Notable differences in the frequency of the *CCR5 Δ32* genotype were observed for baseline CDC category (*CCR5 Δ32/wt* genotype frequency among individuals diagnosed at asymptomatic stage of infection was 22.0% compared to 10.2% among symptomatic and AIDS patients, p<0.003) and overall number of deaths *(the CCR5 Δ32/wt* genotype was observed in 7.5% cases compared to 15.6% of patients surviving throughout the period of observation, p = 0.046). Similar *CCR5 Δ32/wt* genotype frequency was observed among patients with AIDS related deaths (7.6%) while in the group with non-AIDS related death the frequency of the *CCR5 Δ32/wt* genotype was lower (5.0%, p = 0.073 if compared to the entire surviving group). Other differences with borderline significance in the *CCR5 Δ32/wt* genotype distribution included transmission route (16.1%, 11.8%, and 14.2% for homosexual, heterosexual and injection drug use (IDU) related transmissions, respectively, p = 0.07), female vs. male gender (9.7% and 15.7%, respectively, p = 0.08) and initiation of antiretroviral treatment (20.0% among treatment naive individuals vs. 13.0% among cART treated patients, p = 0.09). Majority of women with the *Δ32* allele were infected sexually (n = 9, 64.2%), while among men predominated IDU (n = 32, 56.1%) over sexual transmissions (homosexual route was observed in 18 (31.6%) while heterosexual in 7 cases (12.3%) (p = 0.17). No significant difference between the groups with and without the *Δ32* allele in the overall number of AIDS diagnoses was observed (*the CCR5 Δ32/wt* genotype noted in 15.0% of AIDS free cases vs. 12.4% of patients with the syndrome, p = 0.42) but the AIDS-free time was slightly longer among HIV positive patients with the *CCR5 Δ32/wt* genotype (median (IQR) of 75(19-134) vs. 48 (10-109) months, p = 0.14), despite similar age at diagnosis (median (IQR) for the *CCR5 Δ32/wt* of 29 (25-35) vs. 30 (25-38) years of age for *CCR5 wt/wt*, p = 0.62) and observation time (85 (41-148) for the *CCR5 Δ32/wt* vs. 82 (33-19) months for *CCR5 wt/wt*, p = 0.23).

### Overall mortality and causes of death

**Figure 1:**
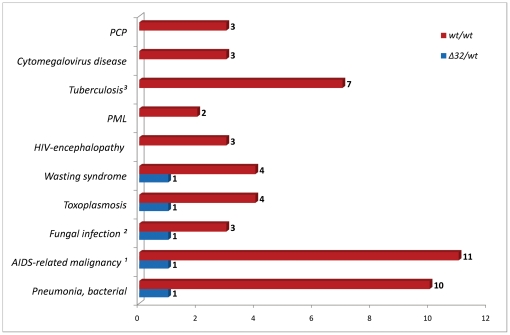
Causes of AIDS-related deaths and *CCR5 wt/wt* vs. *CCR5 Δ32/wt* genotype. ^1^ AIDS-related malignancy: B-cell lymphoma (n = 4), Burkitt lymphoma (n = 1), immunoblastic lymphoma (n = 1), primary CNS lymphoma (n = 4), non-Hodgkin lymphoma - unspecified location (n = 2); ^2^ Fungal infection: pulmonary candidosis (n = 2), generalised cryptococcosis (n = 1); ^3^ Tuberculosis: pulmonary (n = 4), tuberculotic sepsis (n = 3).

**Figure 2:**
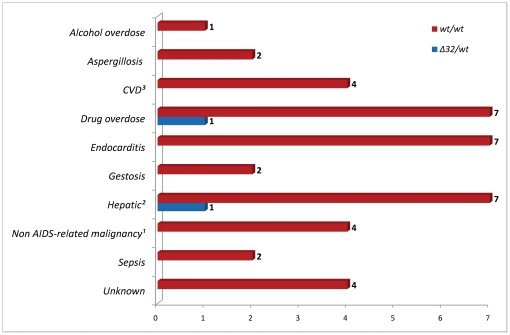
Causes of non AIDS-related deaths and *CCR5 wt/wt* vs. *CCR5 Δ32/wt* genotype. ^1^ Hepatic: liver cirrhosis (n = 5), liver insufficiency (n = 3); ^2^ CVD: stroke (n = 1), coronary artery disease (n = 2), pulmonary embolism (n = 1); ^3^ Non AIDS-related malignancy: Hodgkin lymphoma (n = 1), gastric cancer (n = 1), leukemia (n = 1), testicular cancer (n = 1). Gestosis: death related to pre-eclampsia or eclampsia during pregnancy.

In the group there were 97 deaths observed, 55 (56.7%) being AIDS-related and 42 (43.3%) non-AIDS related. All-cause mortality of 2.66 (95%CI 2.57–3.19) per 100 person-years with AIDS-related mortality of 1.51 (95% CI 1.44–1.90) and non-AIDS related of 1.15 (95% CI 1.09–1.50) per 100 person-years was noted. The most common causes of AIDS related deaths were pneumonia, malignancy and tuberculosis while drug overdose, endocarditis and hepatic events were leading causes of non-AIDS related deaths (figure 1 and 2). In seven patients who died (7.5%) the *CCR5 wt/Δ32* genotype was noted, in majority of these patients (n = 5) AIDS-defining conditions were observed at the time of death while two deaths were non-AIDS related. There were no significant differences for the reasons of death between the groups with and without the *CCR5 Δ32* allele.

### Association between mortality and selected factors

**Figure 3:**
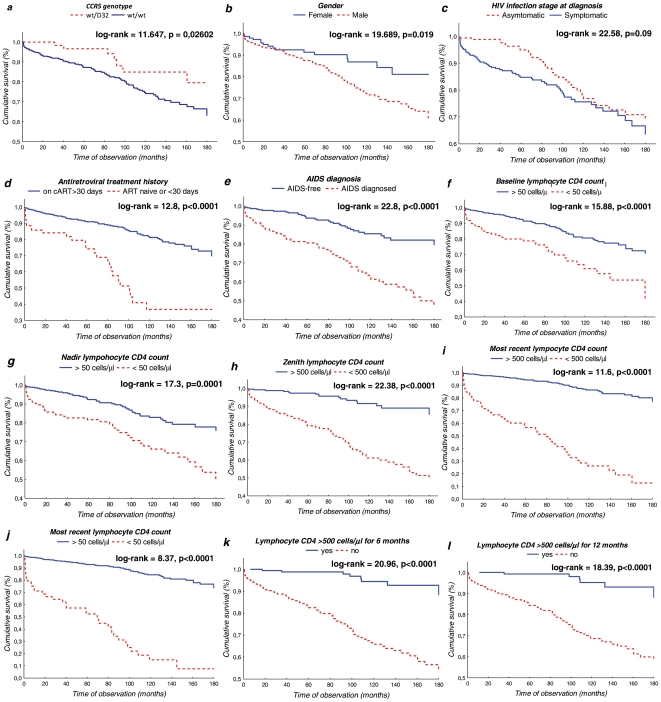
Kaplan-Meyer plots for the most significant parameters. a – *CCR5* genotype, b- gender, c- HIV infection stage at diagnosis, d – antiretroviral treatment history, e- AIDS diagnosis, f – baseline CD4 count of >50 cells/µl, g – nadir CD4 cells >50 cells/µl, h – zenith CD4 >500 cells/µl, i – the most recent CD4 count >500 cells/µl, j- the most recent CD4 count >500 cells/µl, k- CD4 count >500 cells/µl maintained for at least half a year, l - CD4 count >500 cells/µl maintained for at least one year.

Table 2 shows univariate hazard ratios calculated with Cox regression and Kaplan-Meyer cumulative mortality for all parameters investigated in the study. Additionally, for every parameter included, Akaike information criterion (AIC) is presented to indicate the fitness of association with mortality. Identified predictors associated with a significantly greater mortality included the *CCR5* genotype, male gender, symptomatic HIV infection at diagnosis, antiretroviral treatment history and AIDS diagnosis (figure 3a–e). The analysis also included a range of lymphocyte CD4 metrics, namely baseline, nadir, zenith and the most recent CD4 count and time of observation with CD4 above 500 cells/µl. Baseline, nadir and the most recent CD4 counts were further categorized using 100, 50 and 25 cells/µl thresholds; for zenith and time of observation the 500 cells/µl threshold was used. This category was also added in the calculations for the latest CD4 count. The strongest association between the baseline and nadir CD4 counts and mortality was noted for the 50 cells/µl category for baseline and nadir CD4 count (figure 3f–g) as well as CD4 zenith <500 cells/µl (figure 3h) and for patients with the most recent CD4 counts >500 cells/µl (figure 3i). It must be noted that Akaike information criterion for the most recent CD4 was the lowest for 100 cells/µl threshold therefore this value was selected for the multivariate analysis. Two categories were analyzed for time with CD4 count >500 cells/µl vs. patients who have never maintained stably high CD4 counts, namely six and twelve months, respectively (figures 3k,3l). No statistically significant differences were obtained for AIDS and non-AIDS related deaths analyzed separately.

**Table 2:** Unadjusted hazard ratio and risk of death association basing on selected parameters.

Predictor	HR^1^	p (Cox regression)	Lower 95%CI	Upper 95%CI	5-years KM death estimate (%)	10-years KM death estimate (%)	15-years KM death estimate (%)	p value for 15 year KM estimate (log-rank test)	AIC*
***CCR5 genotype***									
*Wt/Δ32*	*reference*				3.23	14.45	20.56	0.026	1090.89
*Wt/wt*	2.28	0.039	1.06	4.92	12.74	25.85	33.57		
***Transmission route***									
IDU	*reference*				10.03	25.76	33.02	0.939	1096.48
SEX	0.98	0.939	0.65	1.48	12.35	20.91	28.98		
***Hepatitis C co-infection status***									
HCV (−)	*reference*				8.28	18.05	28.22	0.566	1096.27
HCV (+)	1.15	0.556	0.72	1.86	9.43	21.65	28.73		
***Gender***									
Female	*reference*				8.60	13.49	19.33	0.017	1090.17
Male	1.90	0.019	1.11	3.25	12.49	28.09	36.12		
***HIV infection stage at diagnosis***									
Asymptomatic	*reference*				4.91	22.94	29.10	0.09	1093.66
Symptomatic	1.43	0.096	0.94	2.18	15.15	24.24	33.55		
***History of antiretroviral treatment***									
cART >1 month	*reference*				8.91	18.47	27.13	<0.0001	1068.35
Never on cART or <1 month	3.83	<0.0001	2.46	5.98	26.11	62.84	62.84		
***Baseline viral load (log copies/ml)***									
VL baseline* >5 log	*reference*				6.49	12.77	18.21	0.526	n/a*
VL baseline <5 log	1.25	0.523	0.63	2.45	10.11	16.55	22.97		
***Baseline lymphocyte CD4 count (cells/µl)***									
CD4 baseline >100	*reference*				7.73	18.91	27.34	0.0002	1082.41
CD4 baseline <100	2.26	0.0001	1.49	3.41	19.07	33.49	41.10		
CD4 baseline >50	*reference*				8.53	19.38	27.67	<0.0001	1081.24
CD4 baseline <50	2.46	<0.0001	1.61	3.77	21.09	38.87	45.79		
CD4 baseline >25	*reference*				9.56	22.68	30.27	0.022	1092.29
CD4 baseline <25	1.76	0.028	1.060	2.920	21.46	29.02	40.21		
***Nadir lymphocyte CD4 count (cells/µl)***									
CD4 nadir >100	*reference*				6.34	17.66	20.87	0.0003	1084.79
CD4 nadir <100	2.17	0.0004	1.42	3.34	16.54	28.06	40.88		
CD4 nadir >50	*reference*				7.51	17.13	22.17	<0.0001	1080.51
CD4 nadir <50	2.50	<0.0001	1.64	3.8	18.33	33.81	46.66		
CD4 nadir >25	*reference*				8.97	20.27	26.81	0.014	1091.46
CD4 nadir <25	1.80	0.014	1.13	2.88	17.55	29.48	42.41		
***Zenith lymphocyte CD4 count (cells/µl)***									
CD4 zenith >500	*reference*				2.60	8.34	10.79	<0.0001	1046.57
CD4 zenith <500	5.51	<0.0001	3.25	9.34	20.79	38.80	48.77		
***Time with lymphocyte CD4 count >500 cells/µl***									
At least 180 days	*reference*				1.15	5.43	7.06	<0.0001	1047.98
Less than 180 days	7.73	<0.0001	3.741	16.00	17.50	34.17	43.80		
At least one year	*reference*				0.71	4.74	6.71	<0.0001	1057.46
Less than one year	7.95	<0.0001	3.47	18.20	15.54	31.32	40.39		
***Most recent lymphocyte CD4 count (cells/µl)***									
Last CD4 >500	*reference*				2.42	7.74	11.26	<0.0001	1062.36
Last CD4 <500	4.84	<0.0001	2.582	9.085	16.33	32.33	41.57		
Last CD4 >100	*reference*				5.73	13.70	19.98	<0.0001	1001.18
Last CD4 <100	9.23	<0.0001	6.128	13.92	43.52	73.93	87.64		
Last CD4 >50	*reference*				7.18	15.61	23.35	<0.0001	1011.89
Last CD4 <50	10.13	<0.0001	6.619	15.51	47.42	84.76	92.38		
Last CD4 >25	*reference*				8.92	19.76	27.03	<0.0001	1051.07
Last CD4 <25	8.48	<0.0001	5.124	14.07	49.46	79.32	100.0		
***History of AIDS***									
AIDS-free	*reference*				6.29	14.66	17.75	<0.0001	1063.16
AIDS-diagnosed	3.30	<0.0001	2.582	9.085	19.45	38.48	50.40		

*For AIC calculations equal group size was used, baseline viral load was excluded from this calculation due to poor availability (288 individuals only).

1 Univariate HR (Hazard Ratio) calculated by unadjusted Cox regression for the same sample sizes as described in the table 1.

### Multivariate survival model with selected parameters

**Figure 4:**
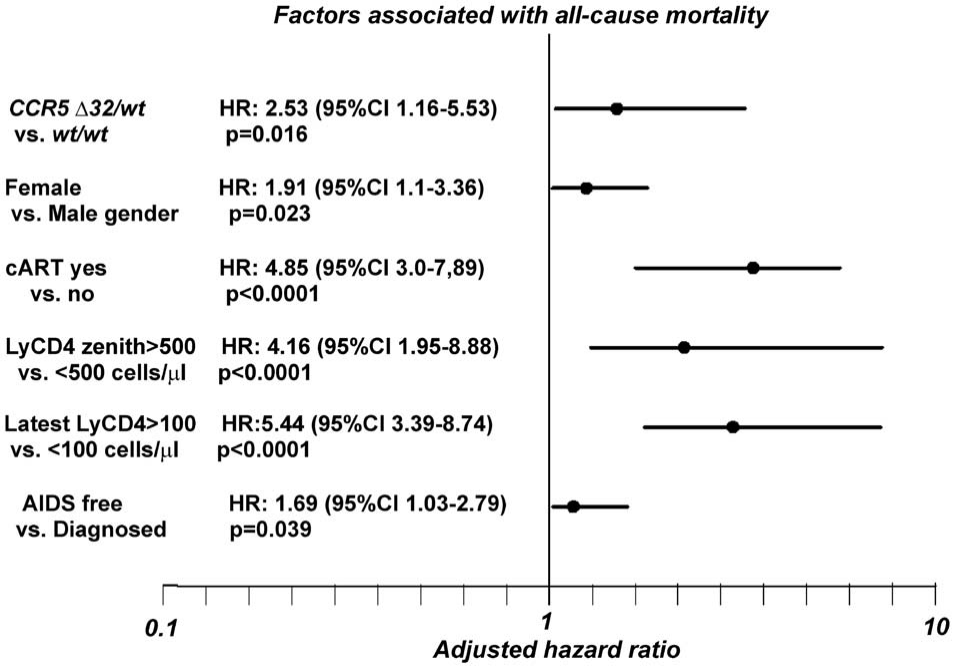
Multivariate Cox regression model of factors modifying 15 year mortality among HIV infected patients (favorable versus unfavorable parameter). Dots represent adjusted hazard ratio (HR) for the unfavorable parameters, lines represent confidence intervals for the HR.

**Figure 5:**
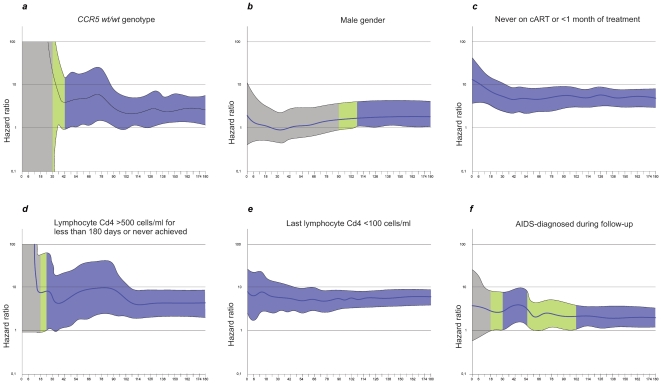
Hazard ratio changes during observation period. For calculation multivariate Cox regression adjusted for the six selected factors was used. Upper and lower confidence intervals are indicated as the external limits in the plot. For the values of borderline significance (p = 0.1–0.50) the field is marked in green while values of statistical significance (P<0.05) are marked in blue. a-*CCR5* genotype, b- gender, c-antiretroviral treatment history, d- CD4 count >500 cells/µl maintained for at least half a year, e-the most recent CD4 count of 100 cells/µl, f- AIDS diagnosis.

Basing on the AIC and statistical significance for each parameter a multivariate Cox model was constructed. In the initial model all parameters presented in table 2 with p≤0.1 for univariate HR were included, for parameters with multiple categories (CD4 metrics) the ones with the lowest AIC were used. Parameters with p<0.1 were removed during analysis prior to construction of the final multivariate model. In the test for the fit of the Cox regression model proportionality was not violated (p values for all selected variables >0.05, with global value of 0.367). In multivariate analysis factors associated with a higher adjusted HR were the *CCR5* genotype (HR for *wt/wt* homozygotes 2.53 (95%CI 1.16–5.53), p = 0.02), gender (HR for males 1.91 (95%CI 1.1–3.36), p = 0.023), introduction of antiretroviral treatment (HR 4.85 (95%CI 3.0–7.89) if untreated or treated for less than one month, p<0.0001, CD4 count of 500 cells/µl for six months (HR 4.16 (95%CI 1.95–8.88) if the parameter was not achieved, p = 0.028), the latest CD4 count (HR 5.44 (95%CI 3.39–8.74) for <100 cells/µl, p<0.0001) and the history of AIDS (HR 1.69 (95%CI 1.03–2.79) for patients with this diagnosis), p = 0.039 (table 3, figure 4). Time-dependent HR plots adjusted for each of the six parameters were drawn to show the effect on survival (figure 5a–f).

**Table 3:** Multivariate model of factors associated with probability of survival in the cohort.

Parameter (favorable vs. unfavorable)	No. (%) of deaths in the group with favorable parameter	No. (%) of deaths in the group with unfavorable parameter	Adjusted HR	Lower 95% CI	Upper 95% CI	Percentage of the risk increase for unfavorable parameter	p value
***CCR5*** ** genotype**							
*Wt/Δ32 vs. wt/wt*	7 (9.8)	86 (19.7)	2.53	1.16	5.53	153%	0.02
**Gender**							
*Female vs. male*	16 (11.1)	81 (22.4)	1.91	1.10	3.36	91%	0.023
**cART >30 days**							
*Yes vs. no*	68 (16.1)	28 (35.0)	4.85	3.0	7.89	385%	<0.0001
**Lymphocyte CD4 >500 cells/µl for 180 days**							
*Yes vs. no*	8 (4.3)	82 (26.1)	4.16	1.95	8.88	316%	<0.0001
**Latest Lymphocyte CD4 count >100 cells/µl**							
*Yes vs. no*	47 (10.9)	47 (64.4)	5.44	3.39	8.74	444%	<0.0001
**Diagnosis of AIDS (ever during follow-up)**							
*No vs. yes*	33 (10.5)	64 (33.2)	1.69	1.03	2.79	69%	0.039

Selection of factors was based on Akaike Information Criterion (AIC) and statistical significance.

### Association between CCR5 Δ32 genotype and survival in treated and untreated individuals

**Figure 6:**
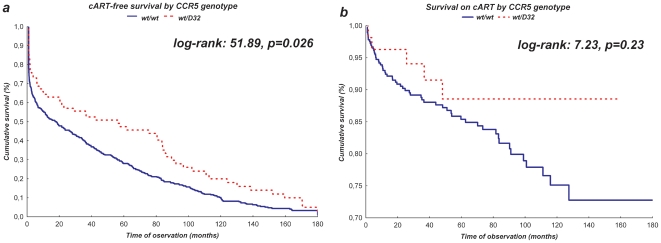
Kaplan-Meyer plots presenting the *CCR5 Δ32/wt* genotype based survival prior to cART introduction (a) and on antiretroviral therapy (b).

To assess the influence of the *CCR5 Δ32* genotype prior to the cART introduction two additional analyses were performed. The first one for the untreated individuals (n = 507 including 71 individuals with the *CCR5 Δ32/wt* genotype), starting from the HIV diagnosis and censored at the time of the cART initiation, loss to follow-up or death (figure 6a). The second one was based on cART treated individuals (n = 422, 55 individuals with the *CCR5 Δ32/wt*, starting from the date of treatment initiation with censoring at the time of termination of data collection, loss to follow-up or death (figure 6b). Among untreated individuals the *CCR5 Δ32/wt* genotype was associated with better survival (p = 0.026), however among cART treated individuals the *Δ32* mutation did not correlate significantly with higher survival rates (p = 0.23).

## Discussion

This study investigates the combined effect of selected parameters and the *CCR5 Δ32* allele on long term survival among HIV-1 infected patients in the era of cART. An array of factors influencing survival were analyzed in this study including demographics as well as clinical and immunological parameters. The frequency of the *CCR5 Δ32/wt* heterozygotes in this study was 14.0% and is consistent to the previous reports for Caucasian populations, while use of archived samples allowed to avoid the bias related to the survival benefit of the mutation in HIV infected patients who may have died [8], [19], [20]. The near-significant difference between the male and female gender frequency might be related to the transmission route – higher number of women was infected sexually. It is possible that the risk of HIV transmission is differently modified by the *Δ32* allele for every gender, depending on the type of exposure.

An association between the *Δ32* allele and delayed progression to AIDS has previously been reported including a suggestion that this deletion is the strongest protective factor for both viral and immununologic control of the HIV-associated disease and improved survival outside the HLA system [10], [21]. In the study by Hendrickson et al. [11], both time to viral suppression and AIDS free survival following cART initiation was significantly longer in the group with the *Δ32* allele. In the meta-analysis by Ioannidis et al., the relative hazard for both AIDS and death was notably lower among the *CCR5 Δ32/wt* heterozygotes if compared to the individuals homozygous for the wild-type variant [22]. The beneficial effect of the *CCR5 Δ32/wt* genotype was also found by Brumme et al. [23], who observed improved survival in the median >5 year period after starting the antiretroviral therapy in the univariate analysis adjusted for baseline age, lymphocyte CD4 count, viral load and AIDS defining conditions.

In our study the number of AIDS-free individuals with the *CCR5 Δ32* variant was significantly higher at the time of HIV diagnosis, however for the entire follow-up period the number of AIDS cases was not notably different between the groups with and without this allele. This could be related to the fact that the cohort used for this analysis consisted of a large number of late diagnosed individuals, in 372 (73.4%) cases infection was diagnosed in either the symptomatic stage or with baseline CD4 count<350 cells/µl, which is characteristic for HIV infections diagnosed in Poland [24]. The protective effect of this deletion may have been lost due to the time-dependency of the beneficial effect, as reported before [25], [26]. Similarly, the slightly higher prevalence of the *Δ32* allele among antiretroviral naive patients is probably related to a higher number of patients asymptomatic at baseline who would not require cART. Other differences of borderline statistical significance for the *CCR5 Δ32* allele frequency include a lower frequency among women, confirming the finding by Philpott et al., of a more extensive protective effect of the *CCR5 Δ32/wt* heterozygous genotype in women [27] while a lower prevalence of the heterozygotes among heterosexually infected patients might be related to the fact that high CCR5 levels are required for these primarily macrophage-tropic sexual infections [28], [29].

In the univariate analysis of all the factors significantly modifying all-cause mortality the strongest protective effect was observed for cART as well as CD4 metrics, namely nadir, zenith lymphocyte CD4, time with CD4 count above 500 cells/µl and AIDS-free observation. Effects of these parameters were extensively studied before and remain well documented [30]–[33]. These influences might be additionally modified by the *Δ32* allele, found also to be a statistically significant protective influence on mortality, as it has been reported that in individuals bearing this variant time of progression to AIDS after initiation of cART as well as time from AIDS diagnosis to death is increased [25], [11]. In our study the *Δ32* allele was associated with decreased mortality risk observed for as long as 15 years of follow-up. This association remained significant after adjustment for other factors strongly influencing mortality: gender, nadir and zenith CD4 count, time with CD4 >500 cells/µl, most recent CD4 levels and AIDS diagnosis.

It must be noted, that the *CCR5 Δ32/wt* genotype was associated with better survival in untreated individuals only, which is probably associated to the slower development of the symptomatic disease as suggested by Brumme et al., [34] while among cART treated individuals the correlation with better survival was insignificant, however this analysis was performed on smaller number of patients (55 with the *CCR5 Δ32/wt* genotype with 23 on cART for 5 years and 6 for more than 10 years). No significant effect of the *Δ32* allele, survival and AIDS free survival was also found by Laurichesse et al. [8]. Moreover, Brumme et al., did not find the association between the *CCR5 Δ32/wt* genotype and time to virological failure in the study of 436 individuals observed for the median follow-up of 22 months [22]. Similarly, Bratt et al, in the study of 147 patients, which included similar (37%) percentage of AIDS diagnosed patients as our analysis, observed little influence of the *CCR5 Δ32/wt* genotype on virologic treatment efficacy [35]. Lack of beneficial effect of the *Δ32* allele among antiretroviral treated patients observed in our study might be associated not only to the size of the group, but also differences in the time from the infection to treatment initiation, which is related to late diagnosis of HIV and high prevalence of AIDS at the time of diagnosis. However, it must be noted that the *Δ32* allele has been associated with the decrease in the likelihood of viral suppression failures, defined as HIV-RNA ≥200 copies/ml at 16–28 weeks of therapy, accelerated viral suppression to <200 HIV-RNA copies/ml, as well as significantly better virologic response to antiretrovirals both at 6 and 12 months following treatment initiation [8], [11], [36]. Moreover, a higher rate of the sustained virologic suppression during the observation period of up to 5 years of antiretroviral treatment in the *CCR5 Δ32*/*wt* heterozygous patients was noted [9].

In adjusted multivariate analyses both AIDS diagnosis and antiretroviral treatment remained significant predictors of mortality. Moreover, it was confirmed that of all the CD4 metrics studied, the latest CD4 count is the strongest predictor of mortality, as has been suggested before [37]. When the time-dependency for the six factors included into the multivariate model was studied, a continuous significant positive effect, regardless the period of observation both for this variable and history of cART, was observed. This confirms the value of these parameters and emphasizes the need for early introduction of the antiretroviral treatment. Stable CD4 count of >500 cells/µl for at least 180 days became a significant parameter after 30 months of observation, which is probably related to the time dependent immune recovery. Interestingly, the continuous protective effect of the *CCR5 Δ32/wt* genotype was observed over 36 months of follow-up with stable HR of 2.5–2.6 noted up to 96 months, the end of observation period. This suggests that in the cART era the protective effect of this variant on survival is not lost over time as was observed for the *Δ32* allele and AIDS diagnosis. Hazard ratio effect plots for gender and diagnosis of AIDS proved time-dependent, with female gender associated with significant protection up to 108 month of observation while for AIDS two periods associated with favorable prognosis in AIDS free patients were noted, namely from months 36 to 54 and 108 to 180 months. The reason for this time dependent relationship remains to be elucidated.

Studies analyzing the impact of the *CCR5 Δ32* deletion on long term survival among HIV (+) patients in the cART era remain sparse, especially with implementation of Cox regression models adjusted for clinical variables significantly contributing to the mortality risk.

A potential limitation of the study was use of interval censoring for six cases when the exact date was unknown, with the death date fixed as the median date between the last observation time and information that the patients actually died. Another limitation is related to the lack of possibility to observe the patients from the time of seroconversion, however in the setting of the late testing and referral of majority of cases with symptomatic immunodeficiency such a study was not feasible. Another limitation is related to the lack of information on adherence, therefore the results could not be controlled for this factor.

Host genetics strongly interacts with HIV leading to the modification of the course of infection. Our finding of the beneficial effect of the *CCR5 Δ32* on long term survival in conjunction with well established factors influencing mortality provides an insight into the interplay between genetic and virus/host-related factors. Such an effect is of the greatest importance in the late-tested populations, such as the one in Poland, allowing for the longer period of delay from infection to disease detection and treatment initiation, whereas it is of the lesser importance in the cART treated individuals. Testing for the *CCR5 Δ32* mutation in clinical practice might be useful for elucidation of the reason for delayed progression of HIV infection, however it should probably not influence decision on antiretroviral treatment initiation or modification.

## References

[1] Bron R, Klasse PJ, Wilkinson D, Clapham PR, Pelchen-Matthews A (1997). Promiscuous use of CC and CXC chemokine receptors in cell-to-cell fusion mediated by a human immunodeficiency virus type 2 envelope protein.. J Virol.

[2] Simon B, Grabmeier-Pfistershammer K, Rieger A, Sarcletti M, Schmied B (2010). HIV coreceptor tropism in antiretroviral treatment-naive patients newly diagnosed at a late stage of HIV infection.. AIDS.

[3] Gonzalez E, Bamshad M, Sato N, Mummidi S, Dhanda R (1999). Race-specific HIV-1 disease-modifying effects associated with *CCR5* haplotypes.. Proc Natl Acad Sci U S A.

[4] Dean M, Carrington M, Winkler C, Huttley GA, Smith MW (1996). Genetic restriction of HIV-1 infection and progression to AIDS by a deletion allele of the CKR5 structural gene.. Science.

[5] Meyer L, Magierowska M, Hubert JB, Rouzioux C, Deveau C (1997). Early protective effect of CCR-5 delta 32 heterozygosity on HIV-1 disease progression: relationship with viral load.. AIDS.

[6] Eugen-Olsen J, Iversen AK, Garred P, Koppelhus U, Pedersen C (1997). Heterozygosity for a deletion in the CKR-5 gene leads to prolonged AIDS-free survival and slower CD4 T-cell decline in a cohort of HIV-seropositive individuals.. AIDS.

[7] Mahajan SD, Agosto-Mojica A, Aalinkeel R, Reynolds JL, Nair BB (2010). Role of chemokine and cytokine polymorphisms in the progression of HIV-1 disease.. Biochem Biophys Res Commun.

[8] Laurichesse JJ, Persoz A, Theodorou I, Rouzioux C, Delfraissy JF (2007). Improved virological response to highly active antiretroviral therapy in HIV-1-infected patients carrying the CCR5 Delta32 deletion.. HIV Med.

[9] Laurichesse JJ, Taieb A, Capoulade-Metay C, Katlama C, Villes V (2010). Is long-term virological response related to CCR5 Delta32 deletion in HIV-1-infected patients started on highly active antiretroviral therapy?. HIV Med.

[10] Ioannidis JP, Contopoulos-Ioannidis DG, Rosenberg PS, Goedert JJ, De Rossi A (2003). Effects of CCR5-delta32 and CCR2-64I alleles on disease progression of perinatally HIV-1-infected children: an international meta-analysis.. AIDS.

[11] Hendrickson SL, Jacobson LP, Nelson GW, Phair JP, Lautenberger J (2008). Host genetic influences on highly active antiretroviral therapy efficacy and AIDS-free survival.. J Acquir Immune Defic Syndr.

[12] Lewden C, Chene G, Morlat P, Raffi F, Dupon M (2007). HIV-infected adults with a CD4 cell count greater than 500 cells/mm3 on long-term combination antiretroviral therapy reach same mortality rates as the general population.. J Acquir Immune Defic Syndr.

[13] Achhra AC, Amin J, Law MG, Emery S, Gerstoft J (2010). Immunodeficiency and the risk of serious clinical endpoints in a well studied cohort of treated HIV-infected patients.. AIDS.

[14] Adler A, Mounier-Jack S, Coker RJ (2009). Late diagnosis of HIV in Europe: definitional and public health challenges.. AIDS Care.

[15] Antinori A, Coenen T, Costagiola D, Dedes N, Ellefson M (2010). Late presentation of HIV infection: a consensus definition.. HIV Med.

[16] (1992). 1993 Revised classification system for HIV infection and expanded surveillance case definition for AIDS among adolescents and adults.. MMWR Recomm Rep.

[17] Rector A, Vermeire S, Thoelen I, Keyaerts E, Struyf F (2001). Analysis of the CC chemokine receptor 5 (CCR5) delta-32 polymorphism in inflammatory bowel disease.. Hum Genet.

[18] Akaike H (1974). A new look at the statistical model identification.. IEEE Trans Autom Control.

[19] Parczewski M, Leszczyszyn-Pynka M, Kaczmarczyk M, Adler G, Binczak-Kuleta A (2009). Sequence variants of chemokine receptor genes and susceptibility to HIV-1 infection.. J Appl Genet.

[20] Guérin S, Meyer L, Theodorou I, Boufassa F, Magierowska M (2000). CCR5 delta32 deletion and response to highly active antiretroviral therapy in HIV-1-infected patients.. AIDS.

[21] Fellay J, Ge D, Shianna KV, Colombo S, Ledergerber B (2009). Common genetic variation and the control of HIV-1 in humans.. PLoS Genet.

[22] Ioannidis JP, Rosenberg PS, Goedert JJ, Ashton LJ, Benfield TL (2001). International Meta-Analysis of HIV Host Genetics. Effects of CCR5-Delta32, CCR2-64I, and SDF-1 3′A alleles on HIV-1 disease progression: An international meta-analysis of individual-patient data.. Ann Intern Med.

[23] Brumme ZL, Henrick BM, Brumme CJ, Hogg RS, Montaner JS (2005). Association of the CCR5delta32 mutation with clinical response and >5-year survival following initiation of first triple antiretroviral regimen.. Antivir Ther.

[24] Bander D, Leszczyszyn-Pynka M, Boroń-Kaczmarska A (2009). [Late AIDS diagnosis in patients hospitalized in Clinic of Infectious Diseases and Hepatology PAM in years 2003–2007].. Przegl Epidemiol.

[25] Meyer L, Magierowska M, Hubert JB, Rouzioux C, Deveau C (1997). Early protective effect of CCR-5 delta 32 heterozygosity on HIV-1 disease progression: relationship with viral load.. AIDS.

[26] Mulherin SA, O'Brien TR, Ioannidis JP, Goedert JJ, Buchbinder SP (2003). Effects of CCR5-Delta32 and CCR2-64I alleles on HIV-1 disease progression: the protection varies with duration of infection.. AIDS.

[27] Philpott S, Weiser B, Tarwater P, Vermund SH, Kleeberger CA (2003). CC chemokine receptor 5 genotype and susceptibility to transmission of human immunodeficiency virus type 1 in women.. J Infect Dis.

[28] Alexander M, Lynch R, Mulenga J, Allen S, Derdeyn CA (2010). Donor and recipient envs from heterosexual human immunodeficiency virus subtype C transmission pairs require high receptor levels for entry.. J Virol.

[29] Wu L, Paxton WA, Kassam N, Ruffing N, Rottman JB (1997). CCR5 levels and expression pattern correlate with infectability by macrophage-tropic HIV-1, in vitro.. J Exp Med.

[30] Neuhaus J, Angus B, Kowalska JD, La Rosa A, Sampson J (2010). Risk of all-cause mortality associated with nonfatal AIDS and serious non-AIDS events among adults infected with HIV.. AIDS.

[31] Lodwick RK, Sabin CA, Porter K, Ledergerber B, Study Group on Death Rates at High CD4 Count in Antiretroviral Naive Patients (2010). Death rates in HIV-positive antiretroviral-naive patients with CD4 count greater than 350 cells per microL in Europe and North America: a pooled cohort observational study.. Lancet.

[32] McKinnon LR, Kimani M, Wachihi C, Nagelkerke NJ, Muriuki FK (2010). Effect of baseline HIV disease parameters on CD4+ T cell recovery after antiretroviral therapy initiation in Kenyan women.. PLoS One.

[33] Gatell JM (2010). When and why to start antiretroviral therapy?. J Antimicrob Chemother.

[34] Brumme ZL, Chan KJ, Dong W, Hogg R, O'Shaughnessy MV (2001). CCR5Delta32 and promoter polymorphisms are not correlated with initial virological or immunological treatment response.. AIDS.

[35] Bratt G, Karlsson A, Leandersson AC, Albert J, Wahren B (1998). Treatment history and baseline viral load, but not viral tropism or CCR-5 genotype, influence prolonged antiviral efficacy of highly active antiretroviral treatment.. AIDS.

[36] O'Brien TR, McDermott DH, Ioannidis JP, Carrington M (2000). Effect of chemokine receptor gene polymorphisms on the response to potent antiretroviral therapy.. AIDS.

[37] Achhra AC, Amin J, Law MG, Emery S, Gerstoft J (2010). Immunodeficiency and the risk of serious clinical endpoints in a well studied cohort of treated HIV-infected patients.. AIDS.

